# Enzymatically Produced Pools of Canonical and Dicer-Substrate siRNA Molecules Display Comparable Gene Silencing and Antiviral Activities against Herpes Simplex Virus

**DOI:** 10.1371/journal.pone.0051019

**Published:** 2012-11-30

**Authors:** Alesia Romanovskaya, Henrik Paavilainen, Michaela Nygårdas, Dennis H. Bamford, Veijo Hukkanen, Minna M. Poranen

**Affiliations:** 1 Department of Biosciences, University of Helsinki, Helsinki, Finland; 2 Institute of Biotechnology, University of Helsinki, Helsinki, Finland; 3 Department of Virology, University of Turku, Turku, Finland; University of Georgia, United States of America

## Abstract

RNA interference (RNAi)-based sequence-specific gene silencing is applied to identify gene function and also possesses great potential for inhibiting virus replication both in animals and plants. Small interfering RNA (siRNA) molecules are the inducers of gene silencing in the RNAi pathway but may also display immunostimulatory activities and promote apoptosis. Canonical siRNAs are 21 nucleotides (nt) in length and are loaded to the RNA Induced Silencing Complex when introduced into the cells, while longer siRNA molecules are first processed by endogenous Dicer and thus termed Dicer-substrate siRNA (DsiRNA). We have applied RNA polymerases from bacteriophages T7 and phi6 to make high-quality double-stranded RNA molecules that are specific for the *UL29* gene of herpes simplex virus (HSV). The 653 nt long double-stranded RNA molecules were converted to siRNA and DsiRNA pools using Dicer enzymes originating from human or *Giardia intestinalis*, producing siRNAs of approximately 21 and 27 nt in length, respectively. Chemically synthesised 21 and 27 nt single-site siRNA targeting the *UL29* were used as references. The impact of these siRNAs on cell viability, inflammatory responses, gene silencing, and anti-HSV activity were assayed in cells derived from human nervous system and skin. Both pools and the canonical single-site siRNAs displayed substantial antiviral activity resulting in four orders of magnitude reduction in virus titer. Notably, the pool of DsiRNAs caused lower immunostimulation than the pool of canonical siRNAs, whereas the immunostimulation effect was in relation to the length with the single-site siRNAs. Our results also propose differences in the processivity of the two Dicers.

## Introduction

RNA interference (RNAi) is an ancient mechanism of gene silencing for diverse eukaryotes [1]. The key components of the RNAi machinery are Dicer and Argonaute endonucleases. Dicer initiates endogenous RNAi by cleaving long double-stranded RNA (dsRNA) molecules into small fragments, referred to as small interfering RNAs (siRNAs) [2]. The siRNAs enter into the RNA Induced Silencing Complex (RISC) with subsequent association of one of the two strands (guide strand) with Argonaute protein, a core component of the RISC. This leads to Argonaute-mediated sequence-specific cleavage of messenger RNA (mRNA) sequence complementary to the bound guide strand [3].

The discovery that exogenous siRNA molecules can trigger the RNAi pathway [4] provided a possibility to generate new therapeutic approaches for the treatment of a wide spectrum of diseases, including genetic disorders, cancer and viral infections [5]. Currently, there are two techniques to generate siRNA molecules for RNAi applications: chemical synthesis [6] and enzymatic production from target-specific DNA templates using RNA polymerases [7]–[10]. In the course of the enzymatic reaction it is possible to synthesize both siRNA [8], [11] and long dsRNA molecules [7], [9]. The latter can be subsequently cleaved *in vitro* by Dicer enzyme generating a pool of target-specific siRNAs representing sequences along the entire region of interest [7], [9], [12], [13]. Although chemical synthesis of single-site siRNAs is the main approach in current RNAi applications, the pools may better maintain their silencing potency in long term usage, in particular when applied to combat viral infections, by reducing the probability of functional escape mutants [14]–[16]. As the concentration of each siRNA species in the pool is low, the pools may also be less potent in inducing off-target effects than the single siRNAs [17].

Depending on the origin, Dicer produces siRNA molecules of slightly different lengths. Human Dicer (HD) generates siRNAs of approximately 21 to 23 nt [9], [18], [19]. These, or equal sized chemically synthesized siRNA molecules, have been widely used for a variety of applications and usually referred to as conventional or canonical siRNA molecules. Dicer from the protozoan parasite *Giardia intestinalis* (GD) cuts dsRNA molecules into longer fragments producing siRNAs of 25 to 27 nt [12], [20]. Although numerous studies indicate that both size classes of siRNA molecules can induce RNAi in mammalian cells [12], [21]–[24] there is still an ongoing debate on the possibility to utilize siRNA molecules longer than canonical siRNAs for therapeutic purposes [12], [23]–[25].

It has been demonstrated [25] that chemically synthesized 27-nt siRNA is a substrate for Dicer enzyme both *in vitro* and *in vivo*. Later it was proved that enzymatically generated 27-nt siRNAs can also be processed by recombinant HD [12]. Therefore, upon introduction into mammalian cells 27-nt RNA duplexes can be recognized and processed by endogenous Dicer potentially advancing siRNA loading into the RISC complex [25]. Accordingly, siRNAs of 27 nt in length, designated as Dicer-substrate siRNAs (DsiRNAs), have been shown to be more efficient in inducing RNAi than the canonical 21-nt siRNAs, especially at subnanomolar concentrations [25]. However, other studies have indicated equal silencing potency for both 21-nt and 27-nt siRNA molecules [12], [21]–[24]. Furthermore, it has been proposed that longer siRNAs are more immunostimulatory than canonical siRNAs in both cell cultures and animals [23], [24]. Longer siRNAs were also shown to induce a substantial decrease in viability of some cell lines [24].

Herpes simplex virus type 1 (HSV-1) is a medically important virus, causing a variety of significant infections, such as oral and skin infections, an increasing proportion of genital herpes, ocular infections, and the rare but severe HSV encephalitis. Single-site siRNAs have been applied to suppress HSV-1 infections in cultured cells using selected target-sequences from the HSV genes encoding VP16 protein and the DNA polymerase [26], glycoprotein E [27], and infected cell protein (ICP) 4 [11], [28]. Furthermore, RNAi has yielded promising results in an animal model of genital herpes (HSV-2) [29] where the single siRNAs used targeted HSV genes *UL29* (a DNA-binding protein, ICP8) and *UL27* (glycoprotein B). Many genes of HSV-1 and -2 have significant homology, and could serve as targets for RNAi.

In the present study we compared the enzymatic activities of HD and GD, evaluated the cellular responses induced by canonical siRNA and DsiRNA molecules and studied the potency of HSV-specific siRNA molecules to block HSV-1 infection in human skin- and nervous system-derived cell lines. For the current study, we chose the prototypic HSV-1 strain 17^+^ as a target, because its genomic sequence is known and it infects well both types of host cells included in our study. The specific aims were to analyze if enzymatically produced siRNA pools could be applied to control HSV infections and to evaluate the possible siRNA length-dependent variation in antiviral and innate immune responses induced by pools of siRNA molecules. Therefore, we created two pools of siRNA molecules, processed either by HD or GD, targeting the *UL29* gene of HSV-1. These were compared to the 21- or 27-nt single-site *UL29*-specific siRNAs or control RNAs representing non-specific sequences. The results suggest that neither canonical nor DsiRNAs influenced cell viability despite a slight up-regulation of interferon pathway genes. Both siRNA pools demonstrated equal potency to suppress virus replication in both cell types, while the 21- and 27-nt single-site siRNAs displayed significantly different immunostimulatory and antiviral activities, especially in the nervous system-derived cell line.

## Methods

### Cell Lines, Plasmids and Bacterial Strains

Transfection experiments were carried out in human glioma U373MG (ATCC) and human epithelial HaCaT [30] cell lines maintained in high glucose Dulbecco’s modified Eagle’s Medium (hg-DMEM, Gibco) supplemented with 2 mM L-glutamine and 10% heat inactivated fetal calf serum (FCS, PromoCell) or in low glucose DMEM (DMEM) supplemented with 7% FCS, respectively. African green monkey (Vero; ATCC) cells were maintained in DMEM supplemented with 2 or 7% FCS. All cells were incubated at +37°C in 5% CO_2_, unless otherwise stated.

The selected target sequence for RNAi within the *UL29* gene of HSV-1 prototype strain 17^+^ (GenBank accession number NC_001806.1) was amplified by PCR using primers containing restriction sites for EcoRI and HindIII (Table S1). The amplified fragment was cloned into the multiple cloning site of pET32b (Novagen) to generate plasmid pET32UL29. Plasmid pCR3.1-eGFP [7] was used to produce an siRNA pool against enhanced green fluorescent protein gene (*eGFP*). Plasmid pLM659 [31] contains the complementary DNA (cDNA) copy of bacteriophage phi6 genome segment S and was used as a template for the production of the 88 base pair (bp) long dsRNA (88-bp dsRNA). *Escherichia coli* strain DH5α was used for propagation of pET32UL29 and pCR3.1-eGFP. Plasmid pLM659 was maintained in *E. coli* JM109.

### dsRNA and siRNA Molecules

The *UL29*- and *eGFP*-specific as well as the 88-bp phi6-specific dsRNA molecules were generated using Replicator RNAi kit (Thermo Scientific) according to the manufacturer’s instructions. For the production of radioactively labeled dsRNA 5 µCi of [α-^32^]UTP (3000 Ci/mmol; Perkin Elmer) was included in the reaction, and unincorporated nucleotides were removed using MicroSpin G-25 Columns (GE Healthcare) after the reaction. DNA templates for dsRNA synthesis reactions were prepared by PCR amplification of the target sequences from an appropriate plasmid using primers containing T7 (TAATACGACTCACTATAGGG) or phi6 (GGAAAAAAA) polymerase promoter sequences at their 5′-ends (Table S1). Enzymatically synthesized dsRNAs were separated from single-stranded RNAs by successive precipitations with 2 M and 4 M LiCl [7]. The resulting dsRNA pellets were washed thoroughly with 70% ethanol and dissolved in RNAse-free Milli-Q water (Millipore).

The synthesized *UL29*- and *eGFP*-specific dsRNA molecules were digested with either GD (PowerCut Dicer, Thermo Scientific) or HD (BLOCK-iT Dicer enzyme, Invitrogen) for 16 h at 37°C according to the manufacturer’s instructions applying 1 U Dicer/µg of dsRNA and dsRNA concentration of 0.2 µg/µl, unless otherwise stated. The reactions containing radioactively labeled dsRNA were stopped at the indicated time points by the addition of 2×U loading buffer (10 mM EDTA pH 8.0, 0.2% SDS, 0.05% bromphenol blue, 0.05% xylene cyanol, 6% (v/v) glycerol, 8 M urea).

For cell culture experiments the desalted Dicer products (NAP column, GE Healthcare) were loaded onto the anion-exchange Gen-Pak FAX column (Waters), connected to the ÄKTApurifier system (GE Healthcare), and eluted (0.5 ml/min) using a linear NaCl gradient in 25 mM Tris-HCI buffer, pH 8.0. Fractions of 0.5 ml were collected and those containing siRNA molecules were combined, desalted with NAP columns, and concentrated using a SpeedVac concentrator (Thermo Savant). Commercial single-site UL29S siRNA (21-nt) [29] and UL29L DsiRNA (27-nt, Table S1) were purchased from Dharmacon and the eGFPS and GAPDH siRNAs were from Qiagen [13]. The single-site siRNAs did not harbor any modifications.

RNA concentrations were determined by measuring the absorbance at 260 nm using a NanoDrop 2000 spectrophotometer (Thermo Scientific). For the evaluation of RNA integrity and purity samples were routinely analyzed by electrophoresis in either 0.8% (dsRNA) or 2.5% (siRNA) agarose gel. The radioactively labeled reaction products were analyzed in 6% polyacrylamide gel in Tris-glycine buffer. After electrophoresis the signals were collected by autoradiography on BAS1500 image plates (Fujifilm), which were scanned using a Fuji BAS-1500 phosphorimager (Fujifilm).

### Transfection and Cell Viability

Enzymatically synthesized siRNA pools, commercial single-site siRNAs, 88-bp dsRNA or water were transfected with Lipofectamine RNAiMAX (Invitrogen) according to manufacturer’s forward transfection protocol using 96-well plates. At the recommended confluency the number of cells per well was approximately 30 000 or 15 000 for HaCaT and U373MG cell lines, respectively. The transfection efficiency was visually assessed using 4 pmol of fluorescein-labeled siRNA (Label IT RNAi Delivery Control, Mirus), and fluorescence was detected with Zeiss AxioVert 200 M microscope. The transfection efficiency was further monitored with 10 pmol of unlabeled human *GAPDH* (glyceraldehyde-3-phosphate dehydrogenase) -specific siRNA (HS_GAPDH_3 FlexiTube siRNA, Qiagen) by measuring changes in the mRNA levels of *GAPDH* gene using real-time quantitative reverse transcription PCR (qRT-PCR; see below). Cell viability was assessed 48 h post transfection with CellTiter-Glo luminescent cell viability assay (Promega) [32].

### Virus Propagation and Titration

Vero cells, grown in roller flasks at +35°C and maintained in DMEM supplemented with 2% FCS, were infected with HSV-1 prototype strain 17^+^ at multiplicity of infection 0.01 and the infection was allowed to spread to the entire culture. The cell debris was removed from the infected culture by centrifugation at 2100×g_avg_, +4°C for 10 minutes. Viruses were collected from the resulting supernatant by centrifugation at 31300×g_avg_, +4°C for 90 minutes. The virus-containing pellet was dissolved in MNT-buffer [20 mM MES (Sigma), 100 mM NaCl, 30 mM Tris 7.4 pH for 24 h in +4°C]. The virus stock was stored in aliquots at –70°C.

HSV-1 titers were determined on Vero cells maintained in DMEM supplemented with 7% FCS and 20 µg/ml human immunoglobulin G using 12-well plates. The plaque forming units (pfu) were counted from infected cells fixed with methanol for 3 min and stained with 0.1% crystal violet in 2% ethanol three days post infection.

RNA-transfected U373MG and HaCaT cells in 96-well plates were infected with 1000 pfu/well of HSV-1 4 h post transfection. Prior to infection cells were washed twice with RPMI 1640 (Gibco) supplemented with 0.1% bovine serum albumin. Infection was performed by addition of 100 µl of medium containing the virus. After 1–1½ h post infection cells were washed three times and finally covered with 200 µl of culture medium per well. 48 h post transfection cells were collected for RNA extraction and the amount of released viruses in the culture medium was determined by plaque titration.

### Quantitative RT-PCR

Total cellular RNA was isolated from cells using TRI Reagent (MRC) according to manufacturer’s instructions. DNase-treated (Fermentas) cellular RNA was reverse transcribed into cDNA using RevertAid H Minus Reverse Transcriptase (Fermentas) and random hexamer primers (Fermentas). cDNA samples were then amplified using Maxima SYBR Green/ROX qPCR Master Mix (Fermentas). qRT-PCR was performed with Rotor-Gene Q real-time instrument (Qiagen) as described earlier [33]. The sequences for the gene specific primers are shown in the Table S1. Relative copy number values of each studied mRNA were obtained by standardization against *GAPDH* mRNA copy numbers in the corresponding sample. In the *GAPDH* knockdown experiment *GAPDH* mRNA levels were standardized against *β-actin* mRNA levels.

### Computational Analyses

Basic Local Alignment Search Tool BLAST [34] and miRBASE [35] were applied in the validation of the siRNA target sequence. Signal intensities of radioactively labeled dsRNA bands were quantitated using 1D Evaluation module of Aida Image Analyzer v. 4.5 software (Raytest Isotopenmeβgeräte GmbH). Statistical analyses were performed using IBM SPSS Statistics 20 software. Mann-Whitney U test was used to calculate significance and threshold was set to p<0.05.

## Results

### Enzymatic Production of siRNA Molecules

#### Selection of the target-sequence within *UL29* gene of HSV-1

For the present work a 653 nt long target-sequence for RNAi was selected from the *UL29* gene coding for the essential ICP8 protein of HSV. The target sequence selection was based on minimal homology with the host genome and maximal homology within to date sequenced HSV strains of types 1 and 2 (GenBank accession number NC_001806.1, GU734771.1, GU734772.1 and NC_001798.1). The selected region (nucleotides 59301 to 59953 of *UL29* gene from HSV-1 strain 17^+^) covers the target-site of the previously used siRNA [29], which we here designate as UL29S (S for small, 21-nt siRNA; Table S1). An extended version of this single-site siRNA was also designed (UL29L; L for long, 27-nt siRNA; Table S1). Non-specific control dsRNA and siRNA molecules were derived from *eGFP* and Pseudomonas phage phi6 genome (88 bp long fragment from the S genome segment; [36]).

**Figure 1:**
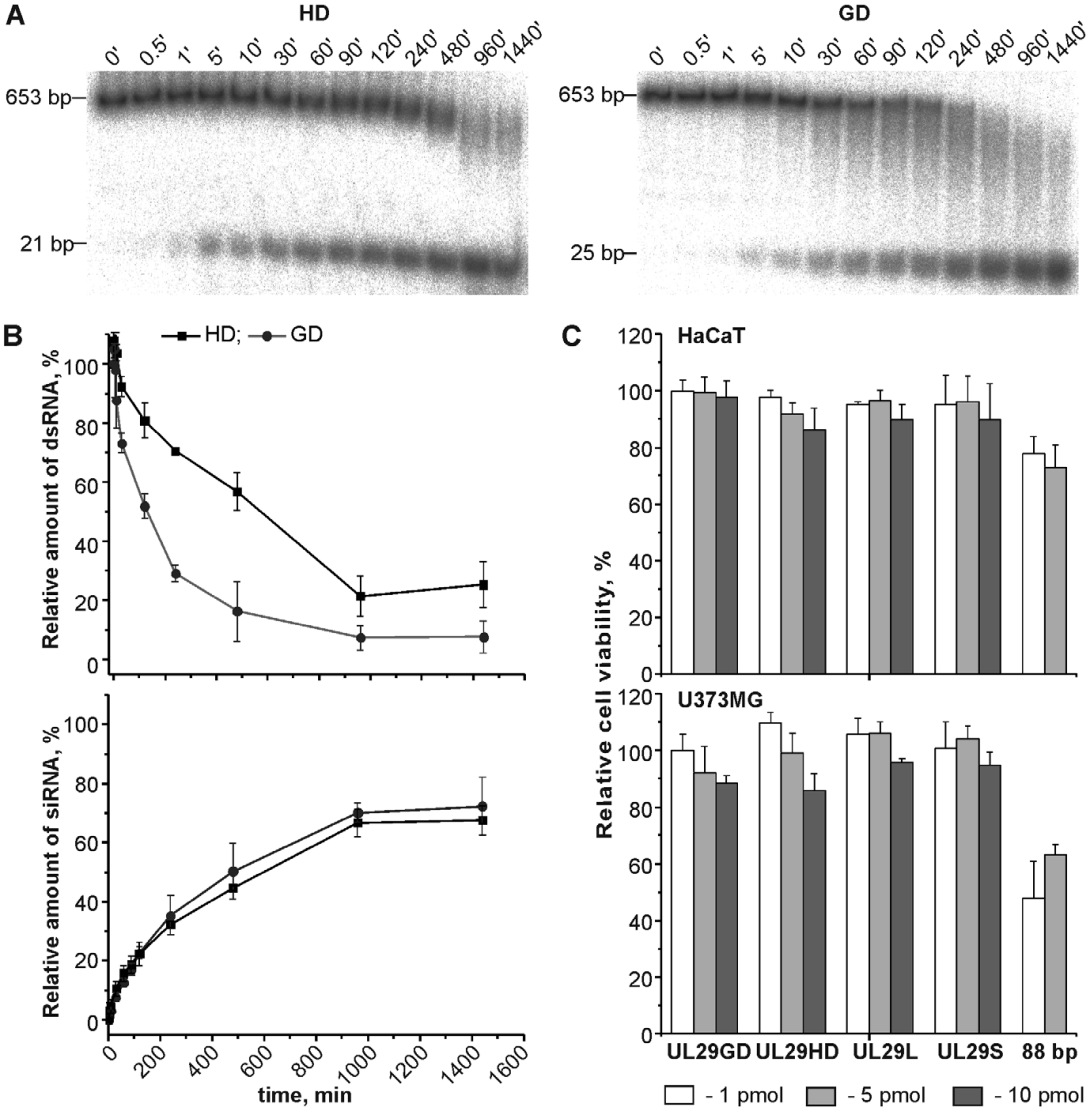
Generation of anti-*UL29* siRNA pools using GD and HD. Internally labeled *UL29* dsRNA was incubated with either HD or GD (1 U of enzyme was used for each µg of dsRNA). (*A*) Samples taken from the HD- (left panel) and GD- (right panel) directed reactions at the indicated time points were analyzed on 6% polyacrylamide gel. The mobility of substrate- and product-length dsRNA molecules is indicated on the left. (*B*) Kinetics of dsRNA substrate processing by HD and GD (upper panel) and time-dependent accumulation of siRNA product in the course of Dicer reaction (lower panel). The dsRNA cleavage (%) (B, upper panel) was calculated as a ratio of the signal intensities corresponding to the full-length dsRNA in each sample and the undigested substrate dsRNA (time point zero). The relative siRNA amount (B, lower panel) is presented as a percentage of the signal intensity of the whole sample. The error bars represent the standard deviation of the mean from two independent experiments. (*C*) Viability of HaCaT (upper panel) and U373MG (lower panel) cells 48 h after transfection with 1 pmol, 5 pmol or 10 pmol of dsRNA molecules. 88-bp dsRNA was applied only in 1 and 5 pmol amounts. UL29GD, GD-digested anti-*UL29* siRNA pool; UL29HD, HD-digested anti-*UL29* siRNA pool; UL29L, 27-nt chemically synthesized anti-*UL29* siRNA; UL29S, 21-nt chemically synthesized single anti-*UL29* siRNA.

The *UL29*-, *eGFP*- and Pseudomonas phage phi6-specific dsRNA molecules were generated from the corresponding DNA (or cDNA) sequences by *in vitro* transcription using T7 DNA-dependent RNA polymerase with subsequent replication of the produced single-stranded RNA molecules by phi6 RNA-dependent RNA polymerase [7]. The purified *UL29*- and *eGFP-*specific dsRNA molecules were afterwards digested to provide siRNA pools using either HD (to obtain 21- to 23-nt siRNAs) or GD (25- to 27-nt siRNAs) enzymes (Figure 1A). The siRNA pools were designated as UL29HD, UL29GD, and eGFPGD.

#### dsRNA processing and siRNA production by GD and HD

The efficiency of dsRNA cleavage and siRNA production by HD and GD was initially evaluated using internally labeled *UL29* dsRNA molecules as a substrate (Figure 1A and B). Aliquots were sampled from HD- and GD-directed reactions at different time points (from 30 s to 24 h) for subsequent electrophoretic analysis (Figure 1A). siRNA-sized molecules were detected already after 1 min and by 16 h (960 min; Figure 1) the reactions were completed. Longer incubation time (up to 24 h) did not result in further decrease in full length dsRNA amount (Figure 1B, upper panel) or increase in siRNA amount (Figure 1B, lower panel). Full-length dsRNA processing by GD was faster than by HD (Fig. 1B, upper panel). However, this did not influence the kinetics of siRNA product accumulation (Fig. 1B, lower panel). The signal for partially processed dsRNA molecules was evidently higher in GD- than in HD-directed reactions (Figure 1A) suggesting that HD possesses higher processivity on a single dsRNA substrate than GD.

#### Enzymatically produced HPLC-purified siRNA pools do not cause toxic drop in cell viability

The produced UL29HD, UL29GD and eGFPGD siRNA pools (as well as the 88 bp long phi6-specific dsRNA fragments) were purified by anion-exchange chromatography with subsequent size exclusion chromatography to desalt RNA samples and remove RNA molecules which are longer than 30 bp or shorter than approximately 10 bp. It is crucially important that purified siRNA pools do not contain traces of undigested or partially digested dsRNA molecules that are longer than 30 bp as such molecules have a strong potency to induce IFN pathways and cellular apoptosis [37]. To verify the quality of the applied purification procedure we transfected human nervous system (glioblastoma-astrocytoma)-derived U373MG and human skin-derived HaCaT (keratinocyte) cells on 96-well plates with 1, 5 or 10 pmol of siRNA pools produced using either GD or HD (Figure 1C). Chemically synthesized single-site siRNAs (UL29S and UL29L) were applied as reference siRNAs. The transfectability of the cell lines was verified with fluorescein-labeled siRNA (data not shown). For quantitative measurements of the transfection efficiency, we transfected a validated commercial GAPDH siRNA into both cell lines and measured the *GAPDH* mRNA knockdown level by qRT-PCR (Figure S1). Transfection of 10 pmol/well of the *GAPDH* siRNA resulted in 98% decrease in GAPDH expression in U373MG cells and in 65% decrease in HaCaT cells (p<0.01 for both cell lines in comparison to control transfections).

**Figure 2:**
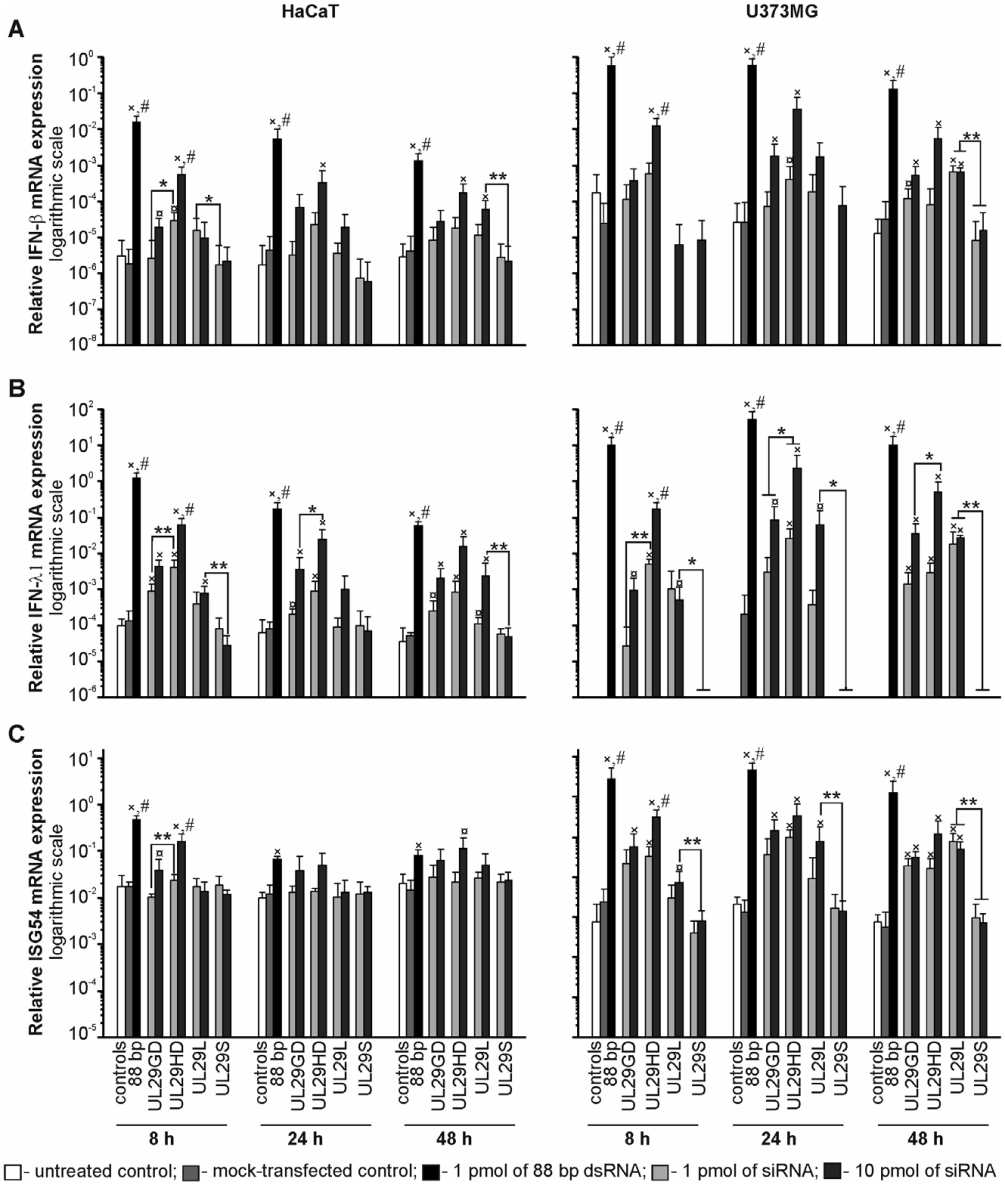
Innate immunity responses to siRNA pools and single-site siRNA molecules. HaCaT and U373MG cells were transfected with either 1 (light grey) or 10 (dark grey) pmol of the indicated siRNA molecules; 1 pmol of 88-bp dsRNA (black bar) or water (control, grey bar); or left untreated (control, white bar). The expression levels of *IFN-β* (*A*), *IFN-λ1* (*B*) or *ISG54* (*C*) were assessed by qRT-PCR 8 h, 24 h or 48 h post transfection. Values were normalized to the *GAPDH* housekeeping gene and shown on a logarithmic scale. The mean values+S.D. of at least two independent experiments, each with a minimum of three biological replicates, are shown. Data were compared by Mann-Whitney U-test. The statistical significance is indicated as (×) p < 0.01 or (¤) p < 0.05 against the controls; (**) p < 0.01 or (*) p < 0.05 against a group of comparison; (#) p < 0.01 against all other transfections. UL29GD, GD-digested anti-*UL29* siRNA pool; UL29HD, HD-digested anti-*UL29* siRNA pool; UL29L, 27-nt chemically synthesized anti-*UL29* siRNA; UL29S, 21-nt chemically synthesized single anti-*UL29* siRNA. No significant signal was detected for IFN-λ1 mRNA in U373MG cell culture (B, right panel) if no dsRNA was applied (non- and mock-transfected cells).

The viability of HaCaT cells did not differ between water- and siRNA-transfected cells (Figure 1C). Likewise, transfection of U373MG culture with 1 pmol of siRNAs did not significantly decrease the number of viable cells. However, at higher siRNA doses the viability of U373MG cells was slightly decreased (<20% reduction). The 88-bp dsRNA caused a clear reduction in the viability of HaCaT cells, and even more prominent effect was observed in U373MG cultures (Figure 1C). This decrease is likely related to the strong activation of interferon responses by the 88-bp dsRNA [36] (see also Figure 2).

### Induction of the Interferon Response by siRNA Molecules

#### Single-site DsiRNA display stronger immunostimulatory activity than its canonical counterpart

To investigate the ability of enzymatically generated and chemically synthesized canonical and DsiRNAs to induce IFN family genes, we treated the cells with either 1 or 10 pmols of different siRNA molecules and analyzed samples from three time points (8, 24 and 48 h) post transfection with qRT-PCR for type I (*IFN-α* and *IFN-β*) and type III (*IFN-λ1*) interferon genes, and interferon-stimulated gene 54 (*ISG54*) expression (Figure 2; data not shown). As a positive inducer of interferon induction we used the 88-bp dsRNA, which is a potent activator of IFN genes with kinetics compatible with the time points of our experiments [36].

As expected, we observed strong activation of *IFN-β*, *IFN-λ1* and *ISG54* genes in response to 88-bp dsRNA with highly significant (p<0.01) difference compared to all other treatments in both HaCaT and U373MG cell lines at almost all time points (Figure 2). The only exception was the relatively low *ISG54* expression observed in HaCaT cells at 24 and 48 h post transfection, which was in accordance with the generally mild *ISG54* response in this cell line (Figure 2C). Additionally, we could observe only minimal alterations in the *IFN-α* gene expression levels in both HaCaT and U373MG cells in all conditions (data not shown).

Canonical single-site siRNA UL29S did not cause IFN responses, whereas the UL29L DsiRNA induced significant dose-dependent activation of most of the measured IFN pathway genes in both cell lines at several time points (Figure 2).

#### The pool of HD-generated canonical siRNAs displays elevated immunostimulatory activity

Both GD- and HD-generated siRNA pools activated *IFN-β*, *IFN-λ1* and *ISG54* expression in a dose-dependent manner (Figure 2). However, after 8 h post transfection the higher dose of HD-produced siRNA pool induced significantly (p<0.01) higher response than the other siRNAs in both cell lines as indicated by the expression level of all the genes studied (Figure 2). The same trend was evident also at the later time points (Figure 2A and C), and in the case of *IFN-λ1* expression the differences between canonical siRNA and DsiRNA pools (UL29HD and UL29GD, respectively) were statistically significant (Figure 2B) at almost all time points.

### Antiviral Activity of siRNA Molecules

#### *UL29*-specific siRNAs induce substantial inhibition of HSV-1 replication

**Figure 3:**
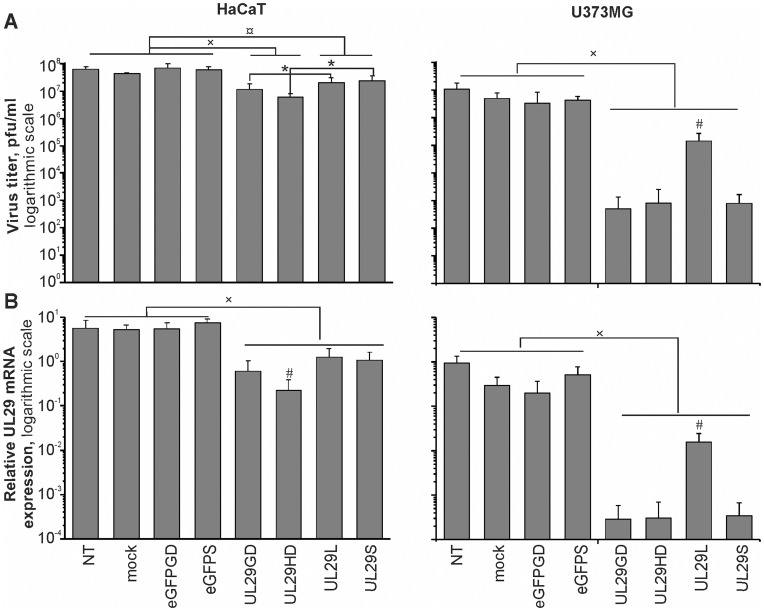
Inhibition of HSV-1 replication by *UL29*-specific siRNA molecules. HaCaT and U373MG cells on 96-well plates were transfected with 10 pmol of indicated siRNA molecules or water. After 4 h the cells were infected with 1000 pfu of HSV-1 and incubated for 44 h. (*A*) Dilutions of the HaCaT and U373MG supernatant, collected 48 h after transfection (44 h post infection) were assayed for released virus by plaque formation on Vero cell culture. (*B*) The relative expression of the target HSV-1 gene *UL29* was measured by qRT-PCR from samples of the infected cultures. Values were normalized to the *GAPDH* housekeeping gene and shown on a logarithmic scale. The mean values+S.D. are shown for six replicates. Data were compared by Mann-Whitney U-test. The statistical significance is indicated as (×) p < 0.01 and (¤) p<0.05 against the controls; (*) p < 0.05 against a group of comparison; (#) p < 0.01 against all other siRNA transfections. NT, no transfection; mock, transfection with water; eGFPGD, anti-eGFP siRNA pool; eGFPS, single-site anti-eGFP siRNA; UL29GD, GD-digested anti-UL29 siRNA pool; UL29HD, HD-digested anti-UL29 siRNA pool; UL29L, 27-nt single-site anti-UL29 siRNA; UL29S, 21-nt single-site anti-UL29 siRNA.

To explore the potential differences in the RNA-dependent gene silencing activity of the HD- and GD-produced siRNA pools (UL29HD and UL29GD) as well as the 21-nt and 27-nt single-site siRNA molecules (UL29S and UL29L), we infected siRNA-treated U373MG and HaCaT cells with HSV-1 strain 17^+^ and determined the viral progeny production by plaque titration (Figure 3A). In addition, we evaluated the effect of the *UL29*-specific siRNA molecules on the expression of the viral target gene by real-time qRT-PCR (Figure 3B). The data obtained demonstrated that all the specific anti-*UL29* siRNA molecules significantly reduced viral shedding and the *UL29* gene expression, whereas the non-specific anti-*eGFP* siRNA molecules (eGFPGD pool and single-site *eGFP*-specific siRNA, eGFPS; Table S1) did not (Figure 3).

#### Anti-*UL29* siRNA molecules demonstrate cell type-specific differences in their ability to inhibit HSV replication

Without siRNA treatment HSV-1 replicated in both cell lines with approximately equal efficiency (Figure 3A). Distinct antiviral effects were observed with all the HSV-specific siRNA molecules. However, the degree of siRNA-induced antiviral effects depended on the cell line used. Treatment of U373MG culture with either GD- or HD-generated *UL29*-specific siRNA pool resulted in a four orders of magnitude reduction in the efficiency of HSV-1 replication, whereas in the HaCaT cells the viral titers were dropped to approximately one-tenth of that observed in the untreated controls (Figure 3A). The lower efficiency of HSV inhibition in HaCaT cells could reflect, at least partially, the differences in transfection competence between the two cell cultures (Figure S1). Furthermore, qRT-PCR experiments revealed that, unlike in U373MG cells, in HaCaT cell culture the anti-*UL29* pool composed of canonical siRNA molecules (UL29HD) caused slightly higher reduction in the *UL29* mRNA levels than the corresponding DsiRNA pool (UL29GD) (Figure 3B). However, these differences in mRNA levels did not result in significant differences in the efficiency of virus replication (Figure 3A).

#### Antiviral effects of *UL29*-specific single-site DsiRNA molecules were less pronounced than the effects of other HSV-specific siRNAs

In the HaCaT cells the antiviral effects of the siRNA pools were more evident than those of the single-site siRNA molecules (Figure 3A); the HaCaT cells transfected with single-site canonical or Dicer-substrate *UL29*-specific siRNA molecules had 2- and 3-fold higher viral titers, respectively, than the cells treated with pools of canonical siRNAs or DsiRNAs, respectively.

In the U373MG cells canonical single-site anti-*UL29* siRNA molecule (UL29S) displayed similar activity as the *UL29*-specific siRNA pools (UL29HD and UL29GD), whereas the antiviral effects of the single-site DsiRNA (UL29L) were less pronounced. Thus, in the U373MG cells treated with single-site DsiRNA molecules, the virus titer decreased only by approximately one order of magnitude instead of four orders of magnitude observed for the other virus-specific siRNA molecules (Figure 3A). The same pattern of siRNA activities was detected also for the expression level of *UL29* gene (Figure 3B).

## Discussion

A variety of approaches have been developed for the selection and production of siRNA molecules for RNAi applications. In this study, we have evaluated antiviral and immunostimulatory activities of canonical siRNA and DsiRNAs pools and compared to those obtained by chemically synthesized siRNAs.

HD and GD processed the 653 bp *UL29*-specific dsRNA with approximately equal efficiency, and both HD- and GD-directed reactions were completed in 16 h (Figure 1A and B). This differs from the earlier studies on HD and GD where shorter substrate molecules were applied and almost 100% cleavage was observed already within 2 h [20], [38]. Comparison of the reaction products produced by the two enzymes revealed differences in the way of dsRNA processing. In GD-directed reaction the amount of full length dsRNA decreased faster than in HD-directed reaction (Figure 1B, upper panel), and dsRNA processing by GD resulted in generation of a pool of intermediate size products, that were not as prominent in HD-catalysed reaction (Figure 1A). Both phenomena indicate that HD has higher processivity on a single dsRNA molecule than GD. GD lacks multiple domains that are present in HD [20]. The lack of one of these domains could promote spontaneous dissociation of GD from the dsRNA substrate molecule between each cleavage step.

Previous studies have indicated that long chemically synthetized siRNA molecules (DsiRNAs) are more potent inducers of interferon pathway genes than the canonical siRNAs [23], [24] and, consequently, it has been proposed that short siRNAs are more safe to use in RNAi applications. Accordingly, we observed stronger induction of all studied interferon pathway genes as a result of transfection with the single-site DsiRNA UL29L than with the canonical siRNA molecule UL29S (27 and 21 nt in size, respectively) (Figure 2). Surprisingly, the effect of the length of the siRNA molecules was different when enzymatically produced siRNA pools were applied. Consequently, it appears that within the range of siRNAs shorter than 30 nt there are other factors than the length of the molecule contributing to the siRNA-induced activation of innate immunity system. The effects of these factors may totally shield the potential effects that are dependent on the size of the molecule. As proposed previously [39], one such factor could be the sequence of the siRNA molecule. In a pool of siRNA molecules the sequence-related effects are averaged due to the low concentration of individual siRNAs, whereas in the single-site siRNA molecules such factors may contribute significantly. Therefore, the results of the effect of the size using the siRNA pools are likely more reliable than those obtained with single-site siRNAs (Figure 2).

The HSV *UL29*-specific siRNAs induced substantial inhibition of viral replication (Figure 3A). The effect was especially prominent when human glioma cells were used, in which four orders of magnitude reduction in virus production was observed. Consequently, RNAi-based approaches could be applied to inhibit HSV infections not only in epithelial cells, as shown previously [29], but also in cells derived from the nervous system, which are the natural hosts for neurotropic viruses such as HSV. The pools of siRNAs appeared especially powerful in viral knockdown (Figure 3A). Inhibition by the canonical single-site UL29S siRNA was also substantial in the glioma-derived cell culture, but the effect of UL29L DsiRNA was modest when compared to all the other *UL29*-specific siRNAs (Figure 3A). Upon the introduction into the cells, DsiRNAs are digested with cellular Dicer enzyme [12], [25] which results in the generation of a set of 21-mer molecules possessing different potency [40]. In the case of the single-site siRNA UL29L, these heterogenic siRNAs demonstrated significantly lower gene silencing activity (U373MG cell line, Figure 3A) although the original 27 bp siRNA comprises the highly efficient UL29S sequence (Table S1). At the same time, a substantial induction of inflammatory responses was observed (Figure 2). Interestingly, the siRNA-induced suppression of HSV replication was equal in cells transfected with HD- or GD-generated siRNA pools presenting canonical and DsiRNAs, respectively. This is in accordance with previous results [12] and implies that the processing of siRNA by endogenous Dicer [25] does not markedly improve the siRNA-induced silencing. Based on earlier studies it was proposed that processing of exogenously applied siRNA by endogenous Dicer could promote siRNA incorporation into the siRNA pathway [25]. However, modified siRNAs that are not processed by Dicer are also incorporated into the RISC [41], and although required in drosophila, Dicer seems to be dispensable for RISC loading in mammals [42]. Apparently, the previously observed differences in the efficiency of siRNA silencing by the two size classes of chemically synthesized siRNAs reflects sequence-dependent variances introduced by Dicer processing [40], which can be difficult to control when using single-site siRNAs.

In conclusion, both HD and GD were equally efficient in the generation of siRNA pools (Figure 1), and siRNA pools produced using HD or GD did not significantly differ in their potency to suppress HSV infection (Figure 3A). Although the enzymatically produced siRNAs, unlike chemically synthesized ones, contained trace amount of siRNA having 5′-triphosphate (one triphosphate in every twelfth or fifteenth siRNA in UL29GD and UL29HD pools, respectively) which are capable of stimulating type I IFN responses [11], transfection of cell cultures derived from human skin or nervous system by either pool resulted only in mild activation of genes involved in interferon pathway, especially when the GD-produced DsiRNA pool was applied (Figure 2). Consequently, both Dicers can potentially be used to produce siRNA molecules for therapeutic applications.

Selection of target sequences is a critical step in the production of single-site siRNA molecules as siRNA with different sequences may have substantially different RNAi activity [43], [44]. Furthermore, the siRNA-induced off-target effects [45] and immunostimulatory activities may differ depending on the selected sequence [39]. Although a variety of siRNA design tools have been developed, not all potentially risky motifs have been discovered [46] bringing additional complexity and unpredictability to the selection process. The difficult design procedure of single-site siRNAs can, however, be largely overcome by using pools of siRNAs, applicable as single-site siRNAs, for example in a topical fashion [29]. The topical delivery of siRNAs may be feasible for treatment of ocular infections caused by HSV-1. Disease models exist for HSV keratitis, where the efficacy and delivery of siRNA pools can be further tested. The siRNA pools may be well suited for antiviral applications, as it is unlikely that functional viral escape mutants or variant viral strains would emerge when the target-sequence is long [14]–[16].

## Supporting Information

Figure S1 **GAPDH knockdown level in HaCaT (A) and U373MG (B) cells.** HaCaT and U373MG cells were transfected on 96-well plate with 10 pmol/well of either GAPDH or eGFPS siRNA. The expression level of GAPDH gene was assessed 48 h post transfection by real-time qRT-PCR. Values were normalized to human β-actin gene. The mean values+S.D. of two independent experiments performed in triplicates are presented. Data were compared by Mann-Whitney U-test. (**) p<0.01. NT, non-transfected control; mock, mock-transfected control; eGFP, cells transfected with non-specific anti-eGFP siRNA; GAPDH, cells transfected with anti-GAPDH siRNA.
(TIF)

Table S1 **Chemically synthesized oligonucleotides used in this study.**
(DOCX)
