# Supplementary figures and images for: Enzymatically Produced Pools of Canonical and Dicer-Substrate siRNA Molecules Display Comparable Gene Silencing and Antiviral Activities against Herpes Simplex Virus
Source: PLoS One. 2012 Nov 30;7(11):e51019. doi: 10.1371/journal.pone.0051019 (PMC3511422; doi:10.1371/journal.pone.0051019)

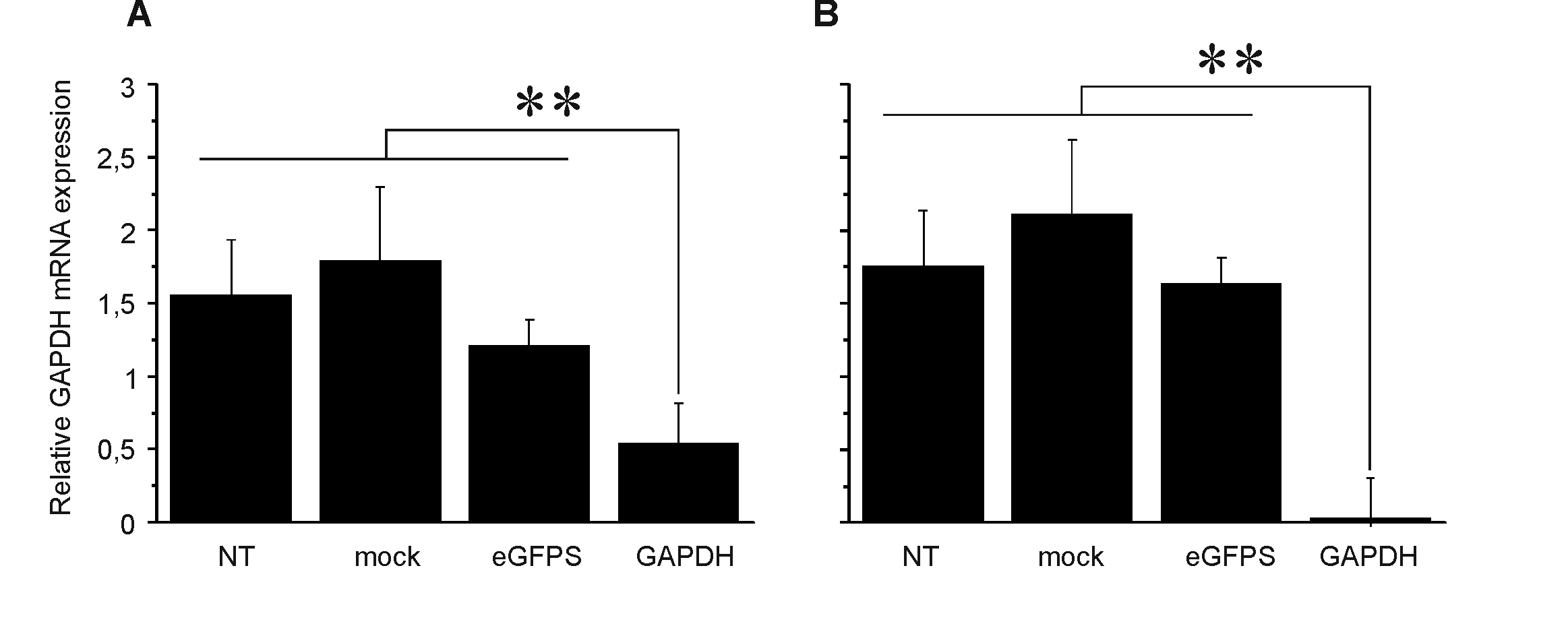

Supplement: Figure S1 — GAPDH knockdown level in HaCaT (A) and U373MG (B) cells. HaCaT and U373MG cells were transfected on 96-well plate with 10 pmol/well of either GAPDH or eGFPS siRNA. The expression level of GAPDH gene was assessed 48 h post transfection by real-time qRT-PCR. Values were normalized to human β-actin gene. The mean values+S.D. of two independent experiments performed in triplicates are presented. Data were compared by Mann-Whitney U-test. (**) p<0.01. NT, non-transfected control; mock, mock-transfected control; eGFP, cells transfected with non-specific anti-eGFP siRNA; GAPDH, cells transfected with anti-GAPDH siRNA. (TIF) [file pone.0051019.s001.tif]
