# Supplementary material for: Enzymatically Produced Pools of Canonical and Dicer-Substrate siRNA Molecules Display Comparable Gene Silencing and Antiviral Activities against Herpes Simplex Virus
Source: PLoS One. 2012 Nov 30;7(11):e51019. doi: 10.1371/journal.pone.0051019 (PMC3511422; doi:10.1371/journal.pone.0051019)
Supplement: Table S1 — Chemically synthesized oligonucleotides used in this study. (DOCX) [file pone.0051019.s002.docx]

**TABLE S1. Chemically synthesized oligonucleotides used in this study.**

| **Name** | **Type** | **Sequence^a^** | **Reference** |
| --- | --- | --- | --- |
| O1_UL29_EcoRI | DNA | 5’‑CGCCGAATTCCGCAACTTTCGCAATCAAT-3’ | this study |
| O2_UL29_HindIII | DNA | 5’‑CGCCAAGCTTATGATGGCCGTAAGGGTGT-3’ | this study |
| O3_UL29_T7 | DNA | 5’-TAATACGACTCACTATAGGGATGATGGCCGTAAGGGTGT-3’ | this study |
| O4_UL29_Phi6 | DNA | 5’-GGAAAAAAACGCAACTTTCGCAATCAAT-3’ | this study |
| O5_eGFP_T7 | DNA | 5’-TAATACGACTCACTATAGGGATGGTGAGCAAGGGCGAGGAG-3’ | this study |
| O6_eGFP_Phi6 | DNA | 5’-GGAAAAAAACTTGTACAGCTCGTCCATGCCG-3’ | this study |
| O7_88bp_T7 | DNA | 5’-CGCGTAATACGACTCACTATAGATAAACAAGTCCTTGTA-3’ | [[1](#_ENREF_1)] |
| O8_88bp_Phi6 | DNA | 5’-GGAAAAAAAGAGAGAGAGCCCCCGAAGG-3’ | [[1](#_ENREF_1)] |
| GAPDH sense | DNA | 5’-GAGAAGGCTGGGGCTCAT-3’ | [[2](#_ENREF_2)]^b^ |
| GAPDH antisense | DNA | 5’-TGCTGATGATCTTGAGGCTG-3’ | [[2](#_ENREF_2)]^b^ |
| ISG54 sense | DNA | 5’-ACTATCACATGGGCCGACTC-3’ | this study^b^ |
| ISG54 antisense | DNA | 5’-TTTAACCGTGTCCACCCTTC-3’ | this study^b^ |
| IFN-λ1 sense | DNA | 5’-GGAGCTAGCGAGCTTCAAGA-3’ | this study^b^ |
| IFN-λ1 antisense | DNA | 5’-GGAAGACAGGAGAGCTGCAA-3’ | this study^b^ |
| IFN-α sense | DNA | 5’-TGGCTGTGAAGAAATACTTCCG-3’ | [[3](#_ENREF_3)]^b^ |
| IFN-α antisense | DNA | 5’-TGTTTTCATGTTGGACCAGATG-3’ | [[3](#_ENREF_3)]^b^ |
| IFN-β sense | DNA | 5’-TCTCCACGACAGCTCTTTCCA-3’ | [[3](#_ENREF_3)]^b^ |
| IFN-β antisense | DNA | 5’-ACACTGACAATTGCTGCTTCTTTG-3’ | [[3](#_ENREF_3)]^b^ |
| HSV-UL29 sense | DNA | 5’-AAGCTGGTTGCGTTGGAG-3’ | this study^b^ |
| HSV-UL29 antisense | DNA | 5’-TTTCTGCTGAAGCAGTTCCA-3’ | this study^b^ |
| β-actin sense | DNA | 5’-TTGCCGACAGGATGCAGAA-3’ | [[4](#_ENREF_4)]^b^ |
| β-actin antisense | DNA | 5’-TCAGGAGGAGCAATGATCATTTGAT-3’ | [[4](#_ENREF_4)]^b^ |
| UL29S | RNA | 5’-CUUUCGCAAUCAAUUCCAAUU-3’  3’-UUGAAAGCGUUAGUUAAGGUU-5’ | [[5](#_ENREF_5)] |
| UL29L | RNA | 5’-ACUUUCGCAAUCAAUUCCAACCGGUGC-3’  3’-UGAAAGCGUUAGUUAAGGUUGGCCACG-5’ | this study |
| eGFPS | RNA | 5’-GCAAGCUGACCCUGAAGUUCAU-3’  3’-GCCGUUCGACUGGGACUUCAAG-5’ | [[2](#_ENREF_2),[6](#_ENREF_6)] |

^a^ The underlined sequences correspond to the indicated restriction endonuclease site or RNA polymerase promoter.

^b^ Annealing temperatures used in qRT-PCR reactions were 60°C for HSV-*UL29*, *IFN-β*, *IFN-λ1 and β-actin* and 55°C for *GAPDH*, *IFN-α* and *ISG54*.

**References**

1. Jiang M, Osterlund P, Sarin LP, Poranen MM, Bamford DH, et al. (2011) Innate immune responses in human monocyte-derived dendritic cells are highly dependent on the size and the 5' phosphorylation of RNA molecules. J Immunol 187: 1713-1721.

2. Nygårdas M, Vuorinen T, Aalto A, Bamford D, Hukkanen V (2009) Inhibition of coxsackievirus B3 and related enteroviruses by antiviral short interfering RNA pools produced using phi6 RNA-dependent RNA polymerase. J Gen Virol 90: 2468-2473.

3. Peri P, Mattila RK, Kantola H, Broberg E, Karttunen HS, et al. (2008) Herpes simplex virus type 1 Us3 gene deletion influences toll-like receptor responses in cultured monocytic cells. Virol J 5: 140.

4. Makela M, Oling V, Marttila J, Waris M, Knip M, et al. (2006) Rotavirus-specific T cell responses and cytokine mRNA expression in children with diabetes-associated autoantibodies and type 1 diabetes. Clin Exp Immunol 145: 261-270.

5. Palliser D, Chowdhury D, Wang QY, Lee SJ, Bronson RT, et al. (2006) An siRNA-based microbicide protects mice from lethal herpes simplex virus 2 infection. Nature 439: 89-94.

6. Caplen NJ, Parrish S, Imani F, Fire A, Morgan RA (2001) Specific inhibition of gene expression by small double-stranded RNAs in invertebrate and vertebrate systems. Proc Natl Acad Sci U S A 98: 9742-9747.
